# Supplementary material for: High prevalence of articles with image-related problems in animal studies of subarachnoid hemorrhage and low rates of correction by publishers
Source: PLoS Biol. 2025 Oct 30;23(10):e3003438. doi: 10.1371/journal.pbio.3003438 (PMC12574824; doi:10.1371/journal.pbio.3003438)
Supplement: S2 Table — The data underlying this Table can be found in https://doi.org/10.5281/zenodo.17192613. (DOCX) [file pbio.3003438.s002.docx]

**Supporting information to “High prevalence of articles with image-related problems in animal studies of subarachnoid hemorrhage and low rates of correction by publishers”, Aquarius et al., PLOS Biology 2025.**

***S2 Table:*** *Overview of suspicious articles that were not labelled as problematic after contact with editors, authors, or careful consideration. The data underlying this Table can be found in https://doi.org/10.5281/zenodo.17192613*

| **Article ID** | **Issue** |
| --- | --- |
| 012 | Article correction: name first author misspelled. PubPeer comment: many figures contained repeated image segments. Found by *René Aquarius*. One of the authors of the study provided the original images, which proved that the publisher edited the figures without the authors’ consent, probably for graphic design reasons. |
| 045 | PubPeer comment: article layout very similar to another, completely unrelated, article. This might suggest origin from the same paper mill or the use of some sort of template. However, there is no conclusive evidence for an error or a serious problem. Found by *René Aquarius*. |
| 120 | PubPeer comment: histological image was used in two articles but only served as an example to illustrate regions of interest (image overlap with Article ID 286). Found by *René Aquarius*. |
| 214 | Article correction: during production, CHOP was defined as *cyclophosphamide, doxorubicin, vincristine, and prednisone* while it should be “*C/EBP homologous protein.*” |
| 278 | PubPeer comment: questions on the use of error bars. Found by *Mortierella elongate*. |
| 286 | PubPeer comment: histological image was used in two articles but only served as an example to illustrate regions of interest (image overlap with Article ID 120). Found by *René Aquarius*. |
| 412 | Article correction: affiliation incorrect for 2 authors. |
