# Supplementary material for: High prevalence of articles with image-related problems in animal studies of subarachnoid hemorrhage and low rates of correction by publishers
Source: PLoS Biol. 2025 Oct 30;23(10):e3003438. doi: 10.1371/journal.pbio.3003438 (PMC12574824; doi:10.1371/journal.pbio.3003438)
Supplement: S3 Table — *Forty-three articles had two countries of affiliation for corresponding authors. **Twenty-one articles had two countries of affiliation for corresponding authors. The data underlying this Table can be found in https://doi.org/10.5281/zenodo.17192613. (DOCX) [file pbio.3003438.s003.docx]

**Supporting information to “High prevalence of articles with image-related problems in animal studies of subarachnoid hemorrhage and low rates of correction by publishers”, Aquarius et al., PLOS Biology 2025.**

***S3 Table:*** *Number of articles per* ***c****ountry of affiliation for corresponding authors. *Forty-three articles had two countries of affiliation for corresponding authors.**Twenty-one articles had two countries of affiliation for corresponding authors. The data underlying this Table can be found in https://doi.org/10.5281/zenodo.17192613*

| **Corresponding author country of affiliation** | **Number of articles (total)** | **Number of articles (problematic)** |
| --- | --- | --- |
| Canada | 2 | 0 |
| China | 453 | 213 |
| Denmark | 1 | 0 |
| France | 1 | 0 |
| Germany | 19 | 2 |
| Iran | 1 | 0 |
| Ireland | 2 | 0 |
| Israel | 1 | 0 |
| Italy | 2 | 0 |
| Japan | 27 | 2 |
| The Netherlands | 5 | 0 |
| Poland | 3 | 1 |
| South Korea | 1 | 1 |
| Sweden | 2 | 0 |
| Switzerland | 4 | 2 |
| Taiwan | 13 | 8 |
| Turkey | 12 | 5 |
| United Kingdom | 1 | 0 |
| United States of America | 101 | 30 |
|  |  |  |
| **Total** | **651*** | **264**** |
