# Supplementary material for: High prevalence of articles with image-related problems in animal studies of subarachnoid hemorrhage and low rates of correction by publishers
Source: PLoS Biol. 2025 Oct 30;23(10):e3003438. doi: 10.1371/journal.pbio.3003438 (PMC12574824; doi:10.1371/journal.pbio.3003438)
Supplement: S1 Fig — Green, dashed bar segments represent nonproblematic articles. Orange bar segments represent problematic articles. Publisher names on the X axis are primarily ranked according to the number of problematic articles. If the number of problematic articles is the same for two or more publishers, the publishers are ordered alphabetically. The data underlying this Figure can be found in https://doi.org/10.5281/zenodo.17192613. (DOCX) [file pbio.3003438.s004.docx]

**Supporting information to “High prevalence of articles with image-related problems in animal studies of subarachnoid hemorrhage and low rates of correction by publishers”, Aquarius et al., PLOS Biology 2025.**


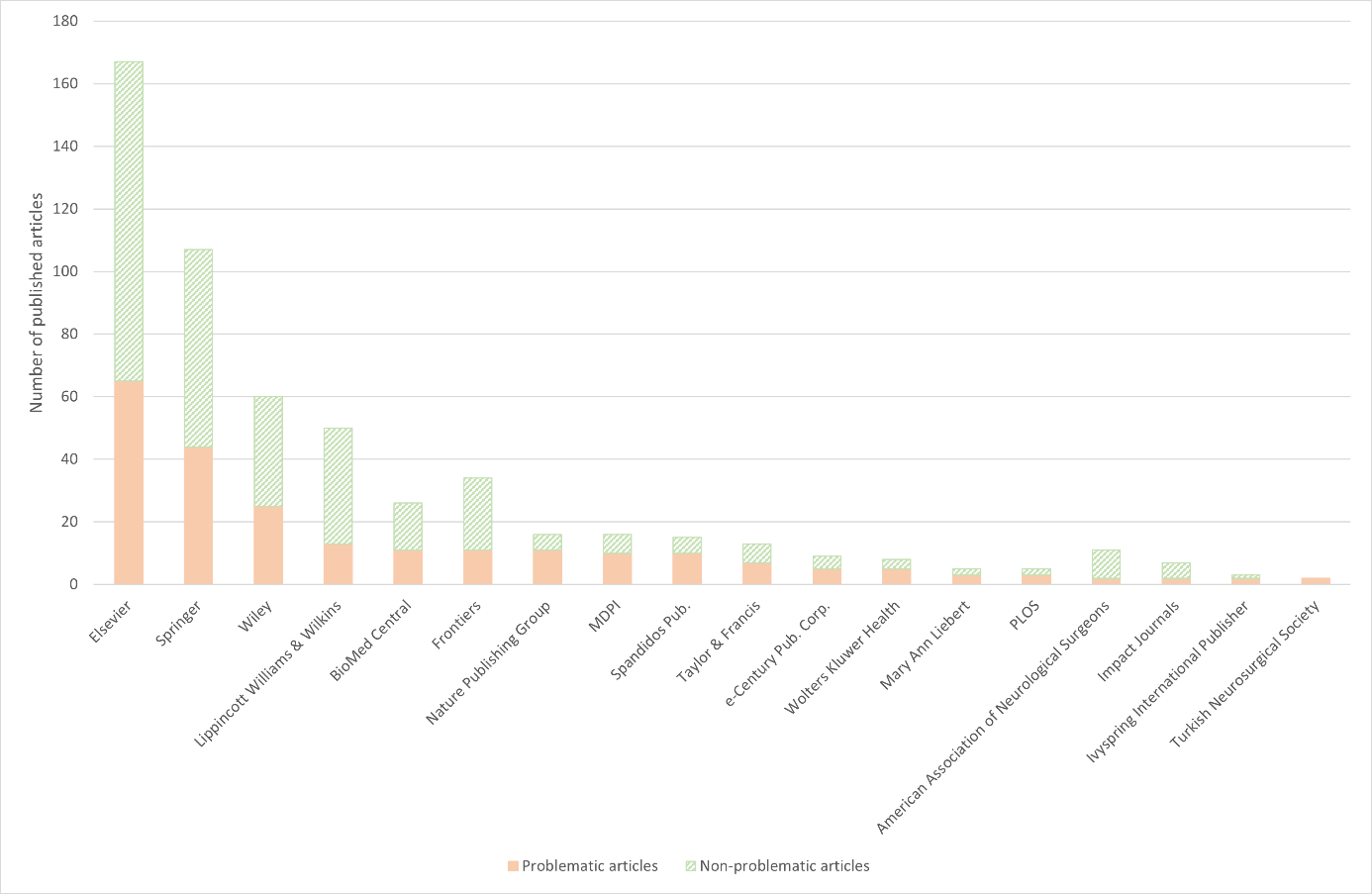


***S1 Fig:*** *Number of problematic articles and non-problematic articles for each publisher that published 2 or more problematic articles. Green, dashed bar segments represent non-problematic articles. Orange bar segments represent problematic articles. Publisher names on the X axis are primarily ranked according to the number of problematic articles. If the number of problematic articles is the same for two or more publishers, the publishers are ordered alphabetically. The data underlying this Figure can be found in https://doi.org/10.5281/zenodo.17192613*
