# Supplementary material for: High prevalence of articles with image-related problems in animal studies of subarachnoid hemorrhage and low rates of correction by publishers
Source: PLoS Biol. 2025 Oct 30;23(10):e3003438. doi: 10.1371/journal.pbio.3003438 (PMC12574824; doi:10.1371/journal.pbio.3003438)
Supplement: Attachment — Submitted filename: 02 Response to the reviewers v2.docx [file pbio.3003438.s007.docx]

Response to the reviewers

Dear dr. Roberts, dear reviewers,

We thank you for the time you have taken to critically review our manuscript. We have taken this opportunity to significantly improve our manuscript and we hope you appreciate the changes we have implemented.

For ease of assessment, we have numbered all comments and inserted comments (when applicable) within the revised manuscript referring to the specific reviewer request.

Once again, thank you for your time and expertise.

Kind regards on behalf of all authors,

René Aquarius

General comments from the academic editor:

**AE01: You'll see that Reviewer #1 is overall positive, but wants you to tone down the language, especially unsupported assertions about the reasons for slow responses from editors. She also suggests mentioning other approaches, to avoid allowing identification of specific individuals, groups or institutions (as this might be legally actionable), and has a list of aspects that need to be substantially tightened up. Reviewer #2 is also positive, but mentions the need for better treatment of the existing literature, has a number of requests for clarification, and asks whether you have permission to quote the editors’ responses.**

*Answer AE01: We value all feedback from the reviewers and have used it to substantially improve the manuscript. All comments have been answered in this document and revisions were implemented where needed.*

**AE02 IMPORTANT: The Academic Editor strongly urges you to tone down your language and confine your interpretation to the evidence that you have to hand, saying "that kind of language runs the risk of turning people off." I also discussed the legal issues with my Editor in Chief, and on her advice we are likely to run the revised version past our legal counsel, so any adjustment of the language should be done in the current round of revision.**

*Answer AE02: Thank you for this advice. We agree the discussion was worded a bit too strongly and we have significantly toned down the language. We hope our revised manuscript presents a more nuanced take and is better aligned with what is (legally) possible.*

**AE03 IMPORTANT: On a personal note, I should say that I have met many editors, from many organisations, and none of them could be described as inept, unwilling or corrupt; indeed they are mostly painfully intent on "doing the right thing." If you have evidence to the contrary, you should present it. Most editors, from my own experience, are simply under-resourced to cope with publication ethics cases on top of their day-job (which I agree is a problem). I've had the interesting experience of working at PLOS both before and after the creation of a dedicated Publication Ethics team; beforehand it was extremely challenging to move ethics cases forward, but this is now handled by the PE team, to everyone's benefit. That said, many cases still involve extensive institutional investigation and legal wrangling, and can take years to resolve... You may find it helpful to interview some professional editors to ask them what *they* perceive to be the barriers to the speedy resolution of publication ethics cases.**

*Answer AE03: We are fully aware of the difficult positions of many academic editors and we have taken a more nuanced approach in our discussion section.*

*R.A.: I have spoken to many professionals in the publishing space in the last two years, ranging from people in the publication ethics departments to academic editors. I am aware of many obstacles the publishing industry is currently facing when curating the scientific record. However, we have also encountered decisions taken by editors that are very difficult to understand. For example stealth corrections (*[*https://onlinelibrary.wiley.com/doi/10.1002/leap.1660*](https://onlinelibrary.wiley.com/doi/10.1002/leap.1660)*). I understand (and acknowledge!) that most editors want to do the right thing. However, I also know the damage a single bad editor can cause. But that is something best discussed in another paper.*

Reviewer #1:

[identifies herself as Jennifer Byrne]

**R1.1: This manuscript describes a significant body of work that was undertaken to examine the integrity of literature on the use of animal models to study preclinical subarachnoid hemorrhage. I should declare that I was aware of this study prior to reviewing the manuscript, for example by inviting the first and senior authors to give a presentation of their preliminary results to a small online group. However, I had not seen this manuscript prior to being invited to review, and I did not know that it had been submitted. While being broadly familiar with the study, I have nonetheless highlighted all issues that I believe might be unclear to the diverse readership that this manuscript is likely to attract.**

*Answer R1.1: Thank you Jennifer for taking the time to comment on our manuscript. I (R.A.) can confirm that Jennifer and I have been in touch on several occasions and that she only knew about preliminary results that we have presented end 2024. In addition I’d like to declare that Jennifer and I are co-authors on this paper (*[*https://doi.org/10.1038/d41586-025-00212-1*](https://doi.org/10.1038/d41586-025-00212-1)*) that was published early 2025.*

*None of the authors have been in contact with Jennifer about this manuscript in any way during the review period.*

**R1.2: The manuscript could also be improved to more clearly describe the full body work that was undertaken, including describing problematic articles to editors and publishers. Some suggestions reflect the required manuscript format, where results are described before the methods. This requires presenting descriptions of basic methods and specifying some other information such as search dates in the results, as the reader won't have encountered this information previously.**

*Answer R1.2: We have improved readability of the manuscript in several ways:*

- *1: With permission of the editor, we have now placed the methods section before the results section, thereby making both sections more clear and succinct..*
- *2: We have included a corresponding author country description in the results section (see also point R1.7)*
- *3: We have removed emphasis on editor an publisher action, as it was not possible to retrieve all relevant email correspondence. This made it impossible to report all relevant dates regarding our correspondence with publishers.*
- *4: We have thoroughly revised the results and discussion sections of the manuscript.*
- *5: We have updated the results on editorial decisions to have an up-to-date overview.*

**R1.3 In some sections, the language should be toned down or changed to be more objective.**

*Answer R1.3: This was suggested by both reviewers and the academic editor and has been addressed by thoroughly revising the discussion section.*

Major issues:

**R1.4: Page 6: This section begins to describe corrective actions by journals and publishers, but it's currently unclear whether corrective actions were in response to the authors' descriptions of their concerns, and/or something else. Text in the methods and results described in the discussion should be moved to the results to make this clear.**

*Answer R1.4: We have now added the requested information and have restructured this information. Due to an update we performed late July, these results have been expanded upon.*

**R1.5: The article includes some expressions and statements that should be toned down or removed: "an overwhelming presence" (page 9, page 15)- this implies that most articles were problematic; "we can confidently say that most journal editors and editors in chief appear to be either inept or unwilling" , "editors are often unaware"; "paper mill bribery may unfortunately be at play here". Please see comments below about potential liability issues.**

*Answer R1.5: We agree that our initial wording was too strong and have thoroughly revised the discussion section and toned down the language considerably.*

**R1.6 Page 9: "We demonstrate their (ie systematic reviews) unique ability to detect problematic articles." (repeated page 15: "unique position"). In fact, at least one other approach identified the same proportions of problematic articles within the human cancer research literature. Our team found 38% of all articles published in the journal Molecular Cancer in 2020 to have wrongly identified gene sequence reagents, and 40% of all miRNA and circRNA articles published in Oncogene in 2020 to have the same problem (Pathmendra P et al. 2024). These proportions are very similar to the 40% of problematic articles identified here, showing that other approaches, ie screening every (or every** **relevant) article in single journals, can generate very similar/ the same results. I therefore suggest removing the word "unique". Much as I'm reluctant to suggest adding citations to our team's work, the similarity between these two sets of results seems worth considering.**

*Answer R1.6: This is a valid point. We have removed the word ‘unique’ from 3 sentences in the discussion and the conclusion of the manuscript. We have also included the suggested reference, which is relevant (!), in our revised discussion.*

**R1.7 Page 9: "across multiple institutions", also "cluster within certain research groups" (page 10)- this data should either be described in the Results or omitted. There could be defamation risks in identifying individual institutions or groups eg. "question the reliability of any research produced by this group" (page 10). It would be easy to identify this group from the articles flagged. It may be safer to discuss papers from a broader perspective, eg. according to country of origin. Perhaps the journal can advise here.**

*Answer R1.7: Identifying information has been completely removed from the manuscript. However, identifying features are still listed in the supplementary excel file (through DOIs, PMIDs, article titles, author names, and PubPeer links). We hope this is acceptable, as this is the only way for readers to verify our results.*

*To enable a more neutral discussion of the demographics of the problematic articles, we have also taken the opportunity to extract the country of the corresponding author(s) for each article. These findings have been added to the methods, results and discussion section. We think this is a big improvement, and thank you for this excellent suggestion.*

**R1.8 Page 18: The literature search was described as having been conducted in February 2023. How did this study then describe problematic articles published in 2024? (Figure 2) The low number of papers identified in 2023 should similarly be discussed, presumably in relation to the screening date.**

*Answer R1.8: Thank you for this suggestion. We understand that this is confusing. We have clarified this in the results section under ‘Study inclusion’ and in the methods section under ‘Study characteristics’. We have also manually double checked all publications from 2023 and 2024 to see if they are still allocated to the correct publication year and this was correct in all cases.*

**R1.9 Finally, I suggest that some consideration to the ethical consequences of problematic papers in terms of research animal use.**

*Answer R1.9: We have included a more in depth reflection on ethical concerns in the revised discussion.*

Minor issues:

**R1.10 Page 1: The current manuscript title is generic- suggest mentioning literature on early brain injury or preclinical subarachnoid hemorrhage.**

*Answer R1.10: We chose a title that better reflects the work presented in the manuscript: “When science fails to self-correct - Problematic articles in a systematic review of animal studies on subarachnoid hemorrhage*

**R1.11 Page 2: "All articles include in a systematic review"… in the abstract, without further context, this suggests that a systematic review was conducted, when this was abandoned. Suggest rewording to make this clear.**

*Answer R1.11: We rewrote the abstract completely. We take the stance that we did conduct a systematic review, but abandoned the planned evidence synthesis of outcome data on early brain injury after subarachnoid haemorrhage. Instead, we have assessed every included article for image-related issues. We have not performed a ‘classic’ meta-analysis with effect size for certain interventions, but have descriptively presented evidence for image-related issues in this body of literature.*

**R1.12 Page 3: "the lack of any clinically effective therapy for early brain injury"- please cite references here.**

*Answer R1.12: We have added the references by Chiara Robba et al. and David C. Lauzier et al. here. We have also done this in another instance where we mention the lack of clinical therapies for early brain injury.*

- *Robba et al. mention early brain injury in their article, but do not explicitly state what can be done to diminish its negative effects, while they have explicitly stated what should be done when other complications after subarachnoid hemorrhage occur.*
- *Lauzier et al. state in their conclusion: “With the occurrence of EBI in patients increasingly measurable, and its underlying mechanisms well-defined, human studies and clinical trials are necessary to identify suitable therapeutics that target this form of secondary brain injury. Active trials in SAH are still primarily focused on reducing DCI, and neglect therapeutic targets relating to EBI.” (EBI stands for Early Brain Injury and DCI for Delayed Cerebral Ischemia)*

*Of note, of the 170 articles Lauzer et al. cites, we flagged 10 for image-related issues. This highlights a real problem when citing relevant literature on the topic: it often cites problematic papers or they are written by authors whose papers we have flagged.*

*For transparency, here are the flagged papers that Lauzier et al. cites:*

1. [*https://pubpeer.com/publications/39A3B3BCFA722CF6F3EDA565277F22*](https://pubpeer.com/publications/39A3B3BCFA722CF6F3EDA565277F22)
2. [*https://pubpeer.com/publications/FFF0003DD593B953A4FAFE02157AA5*](https://pubpeer.com/publications/FFF0003DD593B953A4FAFE02157AA5)
3. [*https://pubpeer.com/publications/9307C74D601B2D6D4D959FF6AB2C88*](https://pubpeer.com/publications/9307C74D601B2D6D4D959FF6AB2C88)
4. [*https://pubpeer.com/publications/D81FB21D163E59241DAAFED1699A2B*](https://pubpeer.com/publications/D81FB21D163E59241DAAFED1699A2B)
5. [*https://pubpeer.com/publications/8A0A30197C521C60F66C30EE04ED28*](https://pubpeer.com/publications/8A0A30197C521C60F66C30EE04ED28)
6. [*https://pubpeer.com/publications/933D0E47DA74E3CEB09414D0A666D5*](https://pubpeer.com/publications/933D0E47DA74E3CEB09414D0A666D5)
7. [*https://pubpeer.com/publications/FA14E4FD68E095A6D3E66BAE9926F7*](https://pubpeer.com/publications/FA14E4FD68E095A6D3E66BAE9926F7)
8. [*https://pubpeer.com/publications/4C9016A64C526CDD6CCA55BE80E7BD*](https://pubpeer.com/publications/4C9016A64C526CDD6CCA55BE80E7BD)
9. [*https://pubpeer.com/publications/3FBEE48228D9977BEA752270560454*](https://pubpeer.com/publications/3FBEE48228D9977BEA752270560454)
10. [*https://pubpeer.com/publications/61788301080FA5E412FF1B82ED6240*](https://pubpeer.com/publications/61788301080FA5E412FF1B82ED6240)

**R1.13: Page 4: "Our comprehensive search"- please provide dates when this was conducted.**

*Answer R1.13: With permission of the editor, we have now placed the methods section before the results section. The reader is now clearly informed when the search took place before they reach the results section.*

**R1.14 Page 4: "The previously defined exclusion criteria"- please describe- readers have not yet read the methods.**

*Answer R1.14: With permission of the editor, we have now placed the methods section before the results section. The reader is now clearly informed what the exclusion criteria are before they reach the results section.*

**R1.15 Page 4: "unflagged"- suggest replacing with "removed from the problematic article group"**

*Answer R1.15: Upon careful consideration, neither the initial wording nor the suggestion is actually correct. We have opted for new terminology: ‘suspicious articles’ and ‘problematic articles’. It would be easier to explain that some suspicious articles would not get the label problematic. We hope you agree with this change.*

**R1.16 Figure 2: Please add details of what's shown on the Y axis.**

*Answer R1.16: We have added an axis title for the Y-axis in the figure.*

**R1.17 Figure 2: Some interesting data are shown but not described or discussed, eg increases in total numbers of papers and problematic papers over time.**

*Answer R1.17: We have now described these findings and their context in more detail in the results section. We have also added a section to the discussion to seek explanations for the rapid growth of the field (see header ‘a growing field’).*

*Again, we want to state here that discussing this topic using the literature is difficult. We feel we have to cite two influential papers (*[*https://doi.org/10.1038/sj.jcbfm.9600283*](https://doi.org/10.1038/sj.jcbfm.9600283) *and* [*https://doi.org/10.1161/STROKEAHA.108.533315*](https://doi.org/10.1161/STROKEAHA.108.533315)*). However, this is not without caveats:*

- *One of the co-authors of both these papers is the author with the most problematic articles we identified so far. The Transmitter recently published about this author in* [*https://doi.org/10.53053/HRDW9635*](https://doi.org/10.53053/HRDW9635)*. However, we have not included this information to avoid potential liability.*

**R1.18 Page 5: "extremely similar"- please define.**

*Answer R1.18: We thought this was a clear case, but apparently it was less clear than anticipated. We have discussed and decided to remove this case as ‘problematic’. To improve the focus of our findings, we have also critically assessed a few other cases and removed those as well. This has all been adjusted under the paragraph “Prevalence of problematic articles” in the results section. This led to some small changes in the data, namely:*

- *The article with the similar layout has been removed from the problematic article list. It has now been added to supporting information file 3: the list of articles that were not labelled as problematic. (*[*https://pubpeer.com/publications/D34D32C75DAF8700B4744AF899E788*](https://pubpeer.com/publications/D34D32C75DAF8700B4744AF899E788)*)*
- *The ‘Lorem Ipsum’ example was removed from the problematic list. However, this article could remain in the problematic list as it also had an image overlap. Upon reviewing the article again, I (RA) remembered that I contacted Elisabeth Bik about this case and that she spotted overlaps that ImageTwin had not picked up. We agreed that she would log these on PubPeer, but she must have forgotten to do it. I therefore did it now and credited dr. Bik for the work she did. (*[*https://pubpeer.com/publications/05E0DCFEEA66F8FDE972DD4A8392CD*](https://pubpeer.com/publications/05E0DCFEEA66F8FDE972DD4A8392CD)*)*
- *We removed an article with a possible western blot splicing issue, as we were unsure of the splicing issue upon review. However, this article did remain in the problematic list, as it has had a correction for a suspicious image duplication in the past (not logged on PubPeer). (*[*https://pubpeer.com/publications/61C11B05A0F5E4506ED0A0601230BC*](https://pubpeer.com/publications/61C11B05A0F5E4506ED0A0601230BC)*)*
- *We added a paper to the problematic category that was retracted by the publisher in June 2025 for image-related concerns. (*[*https://pubpeer.com/publications/5BF22095C4F2FEC8D63761C7965BCE*](https://pubpeer.com/publications/5BF22095C4F2FEC8D63761C7965BCE)*)*

**R1.19 Page 5: "In four articles, we or others"- please indicate which issues were found by the author team, and which were found by others (presumably highlighted on PubPeer?).**

*Answer R1.19: Yes, we mean identifying the initial issue through PubPeer here. We understand the lack of precision associated with this statement. Therefore, for all statements on problematic articles, we calculated the number (and percentage) identified by the author team and added a statement on this in the main text. We have added a column in the ‘problematic’ Excel tab (supporting information file 2) to highlight who found the initial issue for every article, facilitating interested readers to have a look for themselves.*

**R1.20 Figure 3: The legend should describe how the data on the X axis were ranked (according to number of problematic articles?). Please also add details of what's shown on the Y axis.**

*Answer R1.20: This information has now been added.*

**R1.21 Page 6: "how publishers have been affected"- suggest rewording, unclear.**

*Answer R1.21: We have reworded this section.*

**R1.22 Page 7: "almost always"- what proportion exactly?**

*Answer R1.22: This sentence has been removed and the entire segment has been rewritten.*

**R1.23 Page 7: "We decided to log them as an expression of concern"- this is incorrect. Published "expressions of concern" are denoted as such- the article is simply "under investigation". Articles under investigation currently represent a new category of flagged article and it's unclear whether or how these notices resolve over time, ie. an expression of concern may or may not be published as a result.**

*Answer R1.23: This is a good point. We have separated ‘under investigation’ and ‘Expression of concern’ as two different categories.*

**R1.24 Page 9: No references were cited.**

*Answer R1.24: The entire discussion has been thoroughly revised and more literature was cited to put our results in perspective.*

**R1.25 Page 9: "this makes it impossible"- in theory, a systematic review was still possible, with the problematic articles excluded. If this wasn't done, it would be helpful to explain why.**

*Answer R1.25: We have removed this segment from this location and put a reworded version in a more logical place.*

**R1.26 Page 10: Please cite references to support: limitations of image integrity detection methods (line 1), "repeatedly raised by Elisabeth Bik" (line 7).**

*Answer R1.26: There are no references to cite for the limitations of image integrity detection methods. The point we make though, doesn’t need verification from another source, we found image-related issues ourselves through visual inspection while the software did not detect any problems.*

*Regarding the statement by Elisabeth Bik: this has been raised many times by her in presentations. We could, however, not readily find a statement of this in one of her publications. We have therefore removed the attribution to dr. Bik from the discussion, but have kept a description of the issue.*

**R1.27 Page 11: "slow, ineffective and inconsistent": the relevant data were not described in the Results.**

*Answer R1.27: We agree that these data have not been described in enough detail. We therefore have reworked some of the results and the entire discussion has been thoroughly revised and this statement has been removed.*

**R1.28 Page 13: "while communication has significantly improved"- please provide details, since when, how?**

*Answer R1.28: The entire discussion has been thoroughly revised and this statement has been removed.*

**R1.29 Page 14: "the scientific record has been severely compromised"- in what field?**

*Answer 1.29: The entire discussion has been thoroughly revised and this statement has been removed.*

**R1.30 Page 14: "Researchers have likely spent time and resources"- providing citations of problematic papers would be a simple way to strengthen this claim.**

*Answer R1.30: We have added a search on the NIH reporter website to show that tax payer money is actually going to research on the topic of early brain injury following subarachnoid hemorrhage.*

*We have also added an example of the study of Lauzier et al, which was also described in R1.12.*

*We have also slightly reworded the section and have added another hypothesis to point out further problems.*

**R1.31 Page 14: "different detection outcomes"- please outline what these are, otherwise the limitation of mostly using pdf scanning is unclear.**

*Answer R1.31 We have clarified this statement with an example. We think this is much clearer, so thank you for this suggestion.*

**R1.32 Page 14: "individual aspects of the study changed over time"- it may be worth adding something like "and were not always under our control".**

*Answer R1.32: We have used the suggested addition.*

**R1.33 Page 14: "the ImageTwin algorithm kept improving" how was this ascertained? Over what time period was this noted?**

*Answer R 1.33: We have reworded the entire section to provide more clarity.*

**R1.34 Page 15: "multiple re-analyses"- is this described in the Methods?**

*Answer R1.34: Yes, this has been mentioned in the methods section. But we have changed terminology in the discussion to remain consistent.*

**R1.35 Page 16: "title-abstract screening"- if only titles and abstracts were screened, problematic images won't be found. Please clarify.**

*Answer R1.35: Thank you for pointing this out, this was an oversight on our part. We have reworded so it makes sense again.*

**R1.36 Page 17: "reference lists of relevant reviews" please identify/ cite the relevant literature reviews.**

*Answer R1.36: Unfortunately this information was not stored and cannot be reproduced. What we can state is that these reviews were retrieved through title & abstract screening, and we have added this information to the main text.*

**R1.37 Page 17: what constituted an "animal study"- presumably mice and rats? Please specify. This won't be obvious to many readers.**

*Answer R 1.37: We do not restrict our definition of animal study to particular species and used a broad search filter for animal studies to identify studies on early brain injury in any species. In practice, however, we estimate that >95% of the studies are done in rodent models. Because we have not stated any exceptions to the animal filter used in our search, we prefer to keep the wording as is.*

**R1.38 Page 18: "knock-out animals only"- does this mean that articles were excluded if studies only examined knock-out models, and no wild-type models? Again, this won't be obvious to many readers.**

*Answer R1.38: We agree that this wording is confusing. We have therefore added a statement to clarify the reason for the exclusion of knock-out animals is the lack of clinical translatability.*

Reviewer #2:

**R2.1: Manuscript has incredible results and it should be published. The methods are sound. Without any doubt, this manuscript highlights the challenges of paper mills and fraud in science, problems that are significantly underestimated in the scientific community.**

*Answer R2.1: We thank the reviewer for their constructive comments and have altered the manuscript significantly to address the comments of both reviewers and the academic editor.*

Some comments to authors.

**R2.2: „Screening and study inclusion" belong to Methods and Data Selection rather than Results.**

*Answer R2.2: We agree that the title of this subheading is not great and we have renamed it to ‘Study inclusions’.*

**R2.3: A literature review is lacking. Where can this manuscript be placed within the existing literature?**

*Answer R2.3: We have thoroughly revised the discussion section of the manuscript and have included more references so we can better discuss our own results.*

**R2.4: It should be explained what an inappropriate overlap is.**

*Answer 2.4: This has now been explained in the introduction.*

**R2.5: It is unclear what the difference is between "The five journals with the highest incidence of problematic articles were" and "The highest prevalence of problematic articles was found in the following list of journals":**

*Answer R2.5: We agree that this explanation was confusing. We have reworded these sections as follows “The five journals with the highest number of problematic articles” and “The highest percentage of problematic articles was found in the following list of journals”*

**R2.6: What is a problematic article? One that contains image duplication, or are other types of problems included as well?**

*Answer R2.6: With permission of the editor, we have now placed the methods section before the results section. This should make it easier to understand it clear how we defined the concept of ‘problematic article’ before reaching the results section.*

*In short: we have indeed focused on image duplication issues and most as 239 out of 243 problematic articles had image-related problems. 4 Problematic articles had other types of issues that were so blatant that we categorized them as problematic (2 included articles were retracted due to peer-review issues, 1 included article had a table that had significant overlap with a table presented in an article that was not included in our systematic review, and 1 article was corrected because it mentioned the use of an ischemic stroke model in its conclusions instead of the actually used hemorrhagic stroke model.*

**R2.7: It is not clear what "other articles" means. From which sample?**

**"Other articles affected 3 In the process of this review, we also identified 37 problematic articles outside of our included 4 article set."**

*Answer R2.7: These ‘orther articles’ were not originally included in our systematic review, but have been identified to have overlapping images with articles that were included in our systematic review. We have reworded this section in order to make clear where these articles stem from.*

**R2.8: Regarding citations from editors' answers. Was notification or permission from the editors obtained to use the quotes in the publication? Was ethical approval received to send concerns to the journals? This is a suggestion to evaluate the use of quotes from editors' responses, as editors may contact journals to request corrections or retractions of the present article. You may contact Lonni Besancon who has a relevant experience regarding this problem.**

*Answer R2.8: We have thoroughly revised the discussion section and have altered this section specifically. We have omitted the use of direct quotes and have reduced the inflammatory language.*

**R2.9: The manuscript mentions that misconduct could be a reason for the lack of innovation in the field of treatment for hemorrhagic stroke. It would be valuable if the manuscript included more discussion of this issue, as the results are truly shocking.**

*Answer R2.9: We have now further elaborated on this and added more text to the section ‘the cost to society’ in the discussion.*
