# Supplementary material for: High prevalence of articles with image-related problems in animal studies of subarachnoid hemorrhage and low rates of correction by publishers
Source: PLoS Biol. 2025 Oct 30;23(10):e3003438. doi: 10.1371/journal.pbio.3003438 (PMC12574824; doi:10.1371/journal.pbio.3003438)
Supplement: Attachment — Submitted filename: 02 Response to the reviewers revision 2.docx [file pbio.3003438.s008.docx]

Response to the reviewers

Dear dr. Roberts, dear reviewers,

We thank you for your time to review our manuscript again and for your positive feedback on the revisions. We have further fine-tuned our manuscript and we hope you appreciate the changes we have implemented.

For ease of assessment, we have numbered all comments and inserted comments (when applicable) within the revised manuscript referring to the specific reviewer request.

Once again, thank you for your time and expertise.

Kind regards on behalf of all authors,

René Aquarius

General comments from the academic editor:

**AE01: Please change your Title to remove punctuation and make it more appealing. We suggest: "High frequencies of articles with image-related problems in animal studies of brain injury and low rates of correction by publishers"**

*Answer AE01: Thank you for this suggestion. We have reworded your suggestion slightly: "High prevalence of articles with image-related problems in animal studies of subarachnoid hemorrhage and low rates of correction by publishers"*

**AE02: Please attend to the remaining requests from the reviewers.**

*Answer AE02: All remaining comments from the reviewers have now been addressed.*

**AE03: Please re-name your supplementary files “Supporting information file 1,” “Supporting information file 3” and “Supporting information file 4” as “Table S1,” Table S2” and “Table S3.” Rename “Supporting information file 2” as “Data S1” and rename “Supporting information file 5” as “Figure S1.”**

*Answer AE03: We have changed the names of the files as per your request. Because “Supporting information file 2” was moved to Zenodo, we have removed it here.*

**AE04: Please cite the location of the data clearly in all relevant main and supplementary Figure legends, e.g. “The data underlying this Figure can be found in S1 Data” or “The data underlying this Figure can be found in https://zenodo.org/records/XXXXXXXX**

*Answer AE04: We have done this, using the exact wording as suggested here.*

**AE05: Please make any custom code available, either as a supplementary file or as part of your data deposition.**

*Answer AE05: No code has been used in this work.*

**AE06: As you address these items, please take this last chance to review your reference list to ensure that it is complete and correct. If you have cited papers that have been retracted, please include the rationale for doing so in the manuscript text, or remove these references and replace them with relevant current references. Any changes to the reference list should be mentioned in the cover letter that accompanies your revised manuscript.**

*Answer AE06: Our reference list is complete and correct. No cited papers have been retracted at this point. We have removed the reference to a pre-print and have replaced it with the peer-reviewed article that was published just after we submitted the first revised draft of our manuscript.*

Reviewer #1:

[identifies herself as Jennifer Byrne]

**R1.1: I would like to thank the authors for their patience and attention to my many comments on the original submitted version. The revised version is a revelation, in part due to the reordering of the methods and results, which has made the manuscript far more accessible, as well as their extensive rewriting of the text.**

*Answer R1.1: Thank you for your kind words and the encouragement to further improve our manuscript. We have addressed the final minor comments in the text below and in the revised manuscript.*

I only have minor suggestions

**R1.2: Page 6, line 21: "the first 80 articles"- it seems that 80 articles were examined (ie "a random sample of 80 articles"- is there a need to discuss "the first"?**

*Answer R1.2: We do not want to confuse the reader and have reworded the segment to provide more clarity.*

**R1.3: Page 8, line 4: "were reviewed by …. to determine if they were truly problematic" -it would help if a clear definition of problematic articles could be given here, ie "which were defined as….".**

*Answer R1.3: Good suggestion. This has now been added to the text.*

**R1.4: Page 20, lines 2-3: "might be hampered by editors who co-operated…. receiving bribes from paper mills". While I recognise that the language has been toned down considerably, this text seemed relatively strong, particularly as this refers to a small number of incomplete corrections (9 articles, page 19). We have also described incorrect corrections (Byrne et al. 2021, Scientometrics), where we proposed that paper mills might try to manipulate post-publication correction processes. However, this could easily happen without co-operation of the editor. The incorrect corrections that we described probably reflected lack of attention to detail and/or lack of understanding of the principles of molecular biology, that probably applied to both editors and authors. In summary, while the present text could be retained, the language could be softened, ie "might have co-operated….. and/or might be receiving". Otherwise it could imply that the editors of these 9 articles may have been colluding with authors AND receiving bribes, when it's likely that neither took place.**

*Answer R1.4: We have adopted your suggestions in the text.*

**R1.5: Page 20, line 10: please remove "which"- not needed.**

*Answer R1.5: Agreed, “which” has now been removed.*

Reviewer #2:

**R2.1: Authors did significant revision of the manuscript. I would recommend to accept it with minor revisions**

*Answer R2.1: Thank you for the kind words. We have addressed the final minor comments in the text below and in the revised manuscript.*

Some comments

**R2.2: "The study selection procedure is depicted in Fig. 1. Our comprehensive search yielded a total of 2068 references, and an additional 61 were identified from reference lists of relevant reviews. A total of 1276 unique references underwent title and abstract screening, during which 668 articles were excluded using the predefined exclusion criteria."**

**As I understand 2068 papers have been retrieved from EMBASE, etc. I wouldn't call them references because they are papers. References are what is cited in the paper. I suggest to correct and call them papers (articles) to avoid confusion. If they are indeed references, it should be additionally mentioned in methods.**

*Answer R2.2: Nicely spotted! We have updated the text to make it less confusing.*

**R2.3: "A total of 359 figures were affected in one out of 4 ways:"**

**I would precise A total of 359 figures in 239 papers (correct if I'm wrong) were affected in one out of 4 ways:**

*Answer R2.3: Your suggestion has been adopted.*

**R2.4: "First, both visual and AI-driven detection methods have limitations. ImageTwin proved instrumental in identifying image duplications, however, it is not flawless. For example, some problematic articles were identified by eye, as ImageTwin failed to flag any of them."**

**As for limitations, it is important to mention that detection depends on the libraries available (if it is the case in the study. Did you screen against the whole library of ImageTwin?).**

*Answer R2.4: We have added this element in our limitation section of the discussion.*
